# Effects of Central Corneal Stromal Thickness and Epithelial Thickness on Intraocular Pressure Using Goldmann Applanation and Non-Contact Tonometers

**DOI:** 10.1371/journal.pone.0151868

**Published:** 2016-03-21

**Authors:** Marvin Lee, Jaehong Ahn

**Affiliations:** 1 Department of Ophthalmology, DMC Bundang Jesaeng General Hospital, Seongnam, Korea; 2 Department of Ophthalmology, Ajou University School of Medicine, Suwon, Korea; University of Iowa, UNITED STATES

## Abstract

**Purpose:**

To investigate whether corneal thickness parameters measured by optical coherence tomography (OCT), such as central corneal thickness (CCT), central corneal stromal thickness (CCST), and central corneal epithelial thickness (CCET), influence the intraocular pressure (IOP) difference measured by Goldmann applanation tonometry (GAT) and non-contact tonometry (NCT).

**Methods:**

In total, 50 eyes from 50 subjects without glaucomatous defects were included in this retrospective, cross-sectional study. We measured IOP using GAT and NCT and calculated the difference between the two methods. CCT was measured by a Cirrus HD-OCT device using anterior segment imaging. The basement membrane of the epithelium, which was seen as a high-reflection line in the OCT image, was taken as a reference line to measure CCST and CCET.

**Results:**

The mean IOP measured by GAT and NCT was 16.7 ± 3.0 and 18.1 ± 3.8 mmHg, respectively. The mean IOP difference was 1.5 ± 1.7 mmHg, and the IOP measured by NCT was 8.4% ± 11.3% higher than that measured by GAT. The CCET and CCST were 57.9 ± 5.6 and 501.7 ± 33.8 μm, respectively. CCT showed a positive correlation with both GAT IOP (r = 0.648, P < 0.001) and NCT IOP (r = 0.676, P < 0.001). Although CCST showed a significant correlation with GAT IOP and NCT IOP, CCET did not. The difference between GAT IOP and NCT IOP increased with CCT (r = 0.333, P = 0.018), and CCET was positively correlated with the IOP difference between GAT and NCT (r = 0.435, P = 0.002).

**Conclusions:**

IOP increased with greater CCT, and CCST seemed to have a more important role than CCET. CCET also increased with greater CCT, and this may be a possible explanation for the increasing difference in IOP between GAT and NCT with increasing CCT.

## Introduction

Elevated intraocular pressure (IOP) has been determined to be closely related to the development of glaucoma and the progression of glaucomatous damage, based on some well-known clinical trials.[1–3] Thus, precise measurement of IOP is important in the management of glaucoma, as well as in monitoring its progression. Although the most accurate method for this is direct IOP measurement by cannulation, it is impossible to conduct this procedure in a glaucoma clinic. All other methods for IOP measurement are indirect, including the use of Goldmann applanation tonometry (GAT), which is gold standard for IOP measurement. Based on its own principle that GAT measures the force needed to flatten a given area of the cornea, GAT is inevitably affected by central corneal thickness (CCT).[4] In the cannulation study by Ehlers et al.[5], GAT errors were found to be as large as 5 to 6 mmHg in otherwise normal eyes, and GAT appeared to be most accurate with a CCT of 520 μm. Thicker corneas resulted in a higher IOP estimate, while thinner corneas resulted in estimated IOPs lower than the actual value.

Several imaging devices have been developed. CCT can be measured using anterior segment spectral-domain optical coherence tomography (AS-OCT), and several studies have confirmed good reproducibility and repeatability in measuring CCT with AS-OCT; values were comparable with those measured using pachymetry.[6–8] Furthermore, the high-resolution images of AS-OCT allow the examiner to measure central corneal epithelial thickness (CCET) and central corneal stromal thickness (CCST) separately.[9]

Non-contact tonometry (NCT), also known as air-puff tonometry, was originally developed to provide a way to measure the IOP without the need for topical anesthetics; it is widely used in general health screening programs. In previous comparative studies, NCT devices that are reasonably accurate and well correlated with GAT over a range of “physiological” pressures have been reported.[10, 11] Because NCT basically uses the same applanation method (i.e., a standardized puff of air to flatten the cornea), NCT is also affected by CCT.[12, 13]

The main purpose of this study was to investigate whether corneal thickness parameters measured via AS-OCT, such as CCT, CCST, and CCET, influence the IOP differences between NCT and GAT. We also evaluated whether there was any difference in the relationship between CCT parameters and IOP measured using NCT and GAT.

## Materials and Methods

This was a cross-sectional retrospective study in which the medical records of the patients who visited a glaucoma clinic at the Department of Ophthalmology, DMC Bundang Jesaeng General Hospital, from September 2014 to January 2015 were reviewed. All subjects were east Asian (Korean). As part of a general health screening program, subjects were recruited from patients who were referred to our glaucoma clinic from local ophthalmologic clinics for suspected glaucoma and/or high IOP.

This research study followed the tenets of the Declaration of Helsinki and was approved by the local ethical committee of DMC Bundang Jesaeng General Hospital. Written or verbal informed consent was not taken to participate in this study due to it was retrospective study from the medical record of routine clinical procedure. Institutional review board of DMC Bundang Jesaeng General Hospital waived the need for written informed consent from the participants.

**Fig 1:**
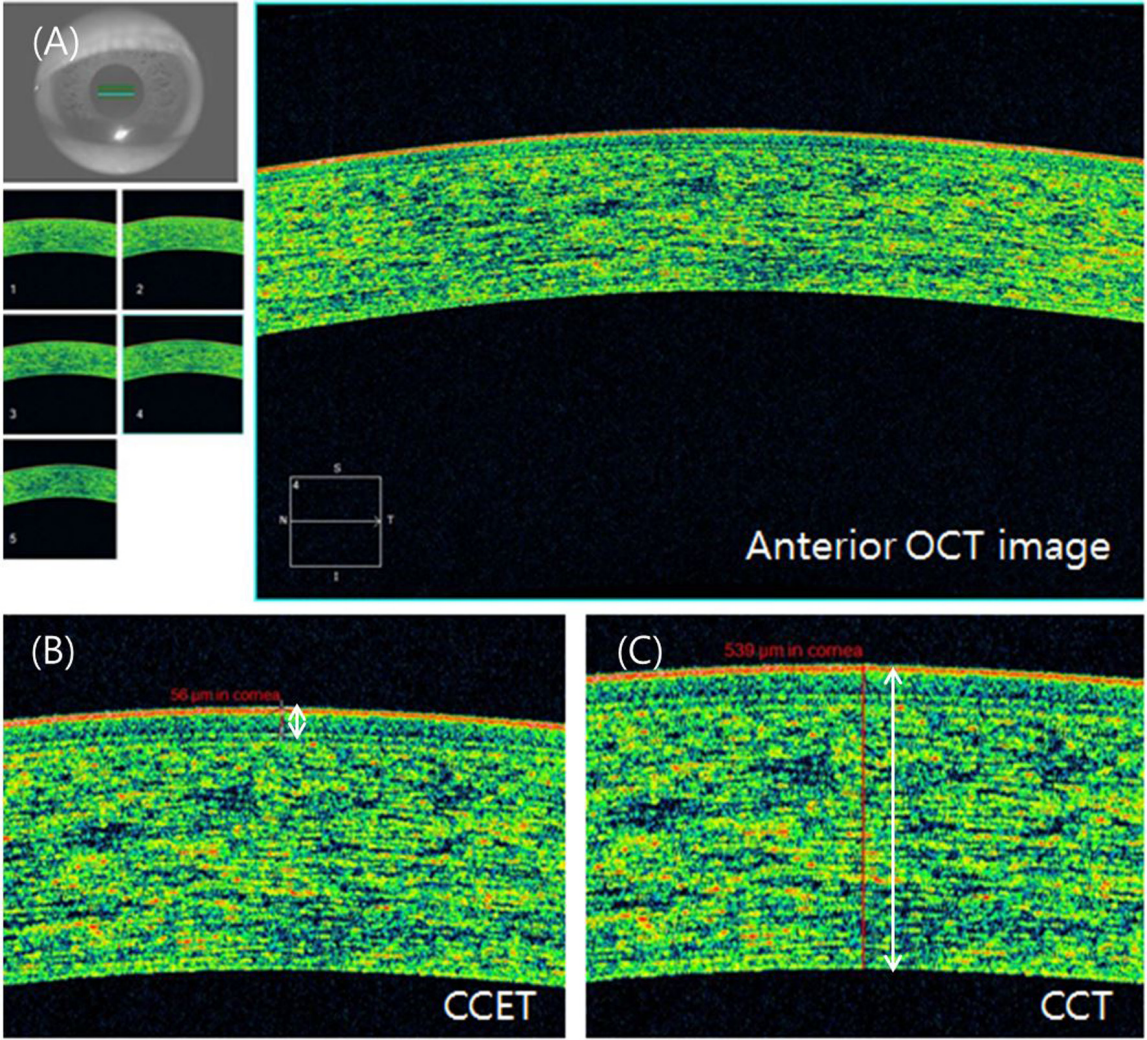
(A) Example of CCT measurement using Cirrus-OCT. (B) CCET was defined as the distance from the air–cornea junction to the Bowman’s layer line (white line). (C) CCT was defined as the distance from the air–cornea junction to the cornea–aqueous humor junction (white line).

Following standard procedures for new referrals to our clinic, all patients underwent comprehensive ophthalmological examinations, including best corrected visual acuity, manifest refraction, stereoscopic optic disc photography, red-free fundus photography (to evaluate the retinal nerve fiber layer), visual field tests, and OCT (Cirrus OCT; Carl Zeiss Meditec, Dublin, CA, USA) for the diagnosis of glaucoma. Patients having any corneal pathologic condition, glaucomatous optic neuropathy and history of previous ocular surgery were excluded. The OCT imaging was performed using Cirrus-OCT software version 4.5. This software allows for the acquisition of a high-definition anterior segment image using the five-line raster scan protocol, which produces five high-resolution (4096 A-scans) horizontal scan lines. Each 1-line raster scan comprises a 3-mm line with an axial resolution of 5 to 6 μm and a transverse resolution of 15 to 20 μm. As described in a previous study, Bowman’s layer was shown as a bright line,[14, 15] as seen in Fig 1. The CCET was defined as the distance from the air–cornea junction to the Bowman’s layer line, the CCST was the distance from the Bowman’s layer line to the cornea–aqueous humor junction, and the CCT was the distance from the air–cornea junction to the cornea–aqueous humor junction. Each thickness was measured via the intrinsic roller function provided in the Cirrus OCT software and defined as the vertical distance between each reference line. We measured each thickness three times from a single OCT scan and used mean values, and the proportion of the stromal thickness to the total corneal thickness (CCSP, Proportion of stromal thickness in central corneal thickness) was calculated from the measured values.

IOP was measured in all patients using GAT and NCT (CT-80 non-contact tonometer; Topcon Corp., Tokyo, Japan) after the patient had been seated for at least 3 min. Typically, IOP measurement were carried out two times with each tonometer in our glaucoma clinic, and the lower value was recorded in the medical record of each patient. The GAT measurement was performed by the same examiner (M.L.); the eyes were anesthetized and a fluorescein strip was applied to the inferior conjunctival fornix. The difference between the GAT and NCT IOPs and the proportional differences in each IOP (CCSP, GAT IOP/NCT IOP) were calculated from the medical records.

Pearson correlation coefficients were calculated to evaluate correlations between parameters. The corneal thickness parameters (CCT, CCST, and CCET) were investigated in linear regressions to determine which factors most influenced the IOP differences between GAT and NCT. All analyses were performed using the SPSS software (ver. 18.0 for Windows; SPSS, Inc., Chicago, IL, USA). A P value of <0.05 was considered to indicate statistical significance.

## Results

In total, 50 eyes (42 with normal, 8 with ocular hypertension) of 50 patients were included in the study. The mean age of the subjects was 49.2 ± 13.1 (range, 19–76) years, and the mean IOPs measured using GAT and NCT were 16.68 ± 3.05 and 18.12 ± 3.84 mmHg, respectively. The CCT, CCET, and CCST, measured using SD OCT were 559.6 ± 36.0, 57.9 ± 5.6, and 501.7 ± 33.8 μm, respectively (Table 1). CCET was increased with increasing CCT (r = 0.443, P = 0.001, simple linear regression analysis).

**Table 1:** Descriptive statics of involved patients.

	Mean	Standard deviation	Minimum	Maximum
Age	48.2	11.0	19	70
BCVA	0.976	0.056	0.8	1.0
Cylinder	-0.552	0.567	-1.500	1.150
Spherical Equivalent	-1.561	2.103	-5.875	1.825
GAT IOP	16.7	3.0	10	23
NCT IOP	18.1	3.8	9	27
GAT IOP—NCT IOP	1.5	1.7	-2	5
CCT	559.6	36.0	490	630
CCET	57.9	5.6	46	71
CCST	501.7	33.8	440	571
CCSP	0.896	0.009	0.874	0.915

BCVA, best corrected visual acuity; GAT, Goldmann applanation tonometry; IOP, intraocular pressure; NCT, Non-contact tonometry; CCT, Central corneal thickness; CCET, Central corneal epithelial thickness; CCST, Central corneal stromal thickness; CCSP, Proportion of stromal thickness in central corneal thickness.

**Fig 2:**
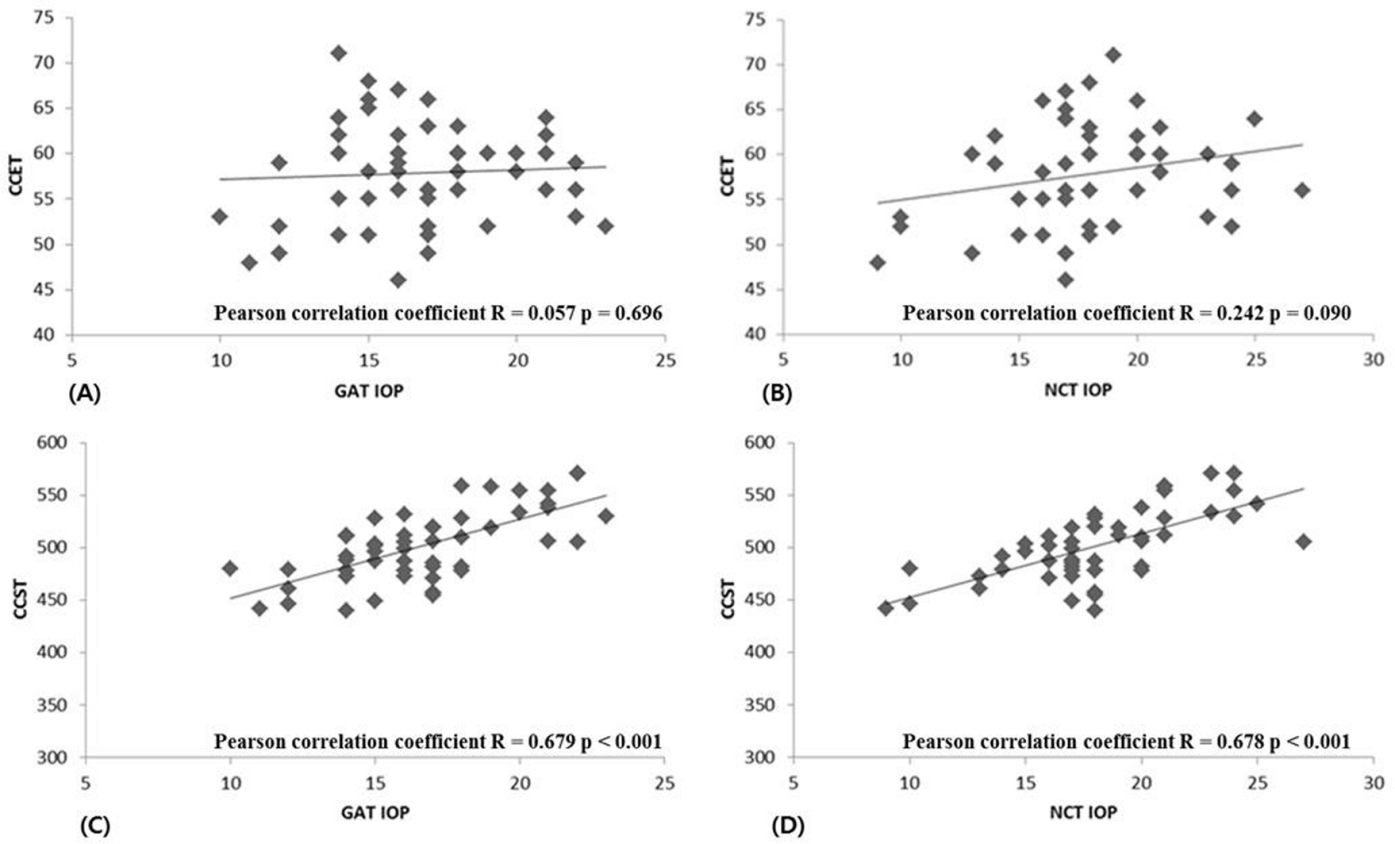
(A) Correlation between CCET and IOP measured by GAT. (B) Correlation between CCET and IOP measured by NCT. (C) Correlation between CCST and GAT IOP. (D) Correlation between CCST and NCT IOP.

**Fig 3:**
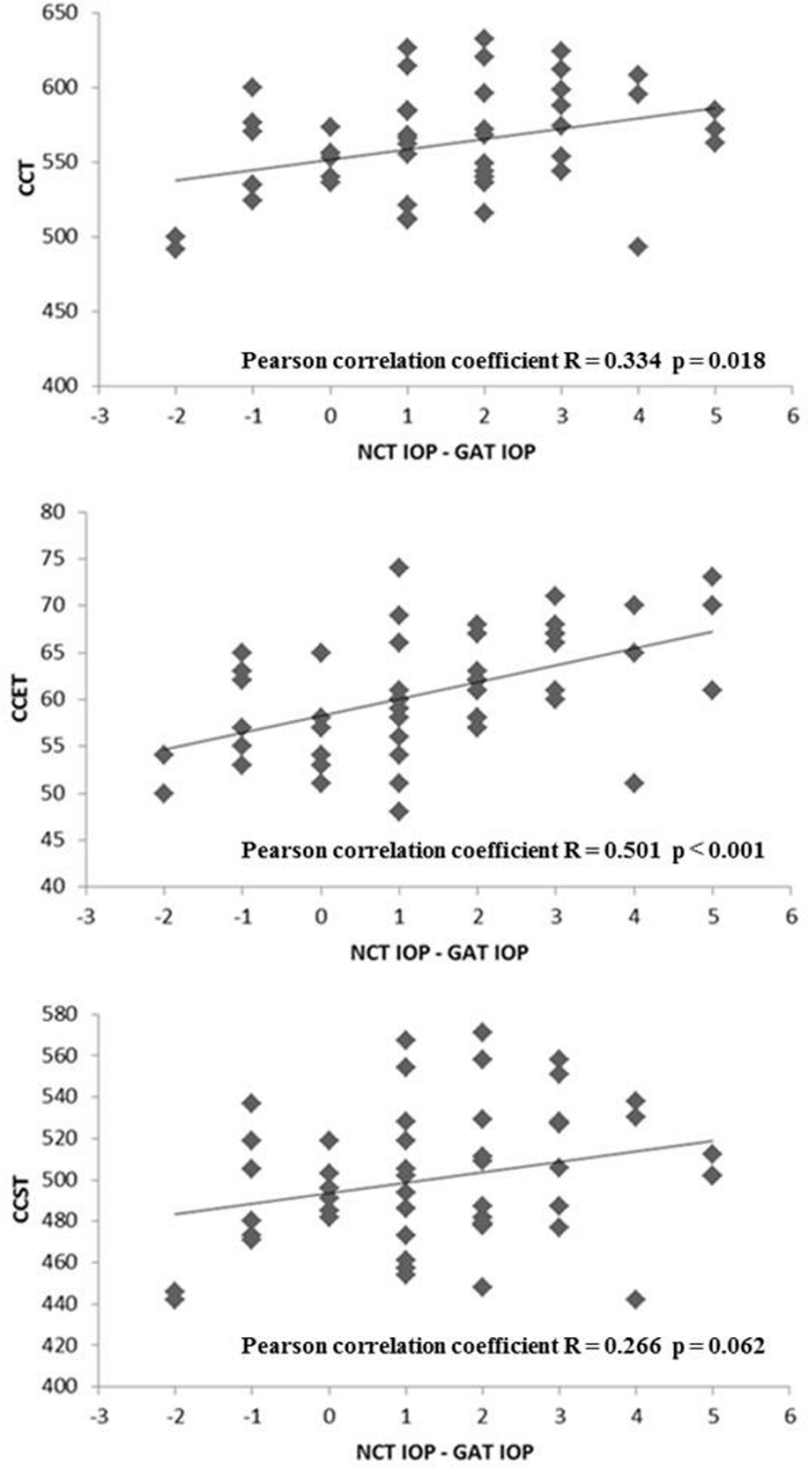
(A) IOP difference between CCT and IOP measured by GAT and NCT. (B) Correlation between GAT IOP and NCT IOP difference and CCET. (C) Correlation between GAT IOP and NCT IOP difference and CCST.

As shown in Table 2, CCT correlated well with GAT IOP, NCT IOP, and the IOP difference between GAT and NCT. CCET did not correlate with GAT IOP (r = 0.057, P = 0.696) or NCT IOP (r = 0.242, P = 0.090), but it did correlate with the IOP difference between GAT and NCT (r = 0.435, P = 0.002). CCST correlated with both GAT IOP (r = 0.679, P < 0.001) and NCT IOP (r = 0.678, P < 0.001), but not with the IOP difference between GAT and NCT (r = 0.281, P = 0.051). Furthermore, CCSP was negatively correlated with the IOP difference between GAT and NCT (r = –0.239, P = 0.095) (Table 2, Figs 2 and 3). Multiple linear regression analyses were performed to evaluate the factors associated with the IOP differences between GAT and NCT using CCT, CCET, CCST, age, and SE; only CCET showed a significant correlation (r = 0.435, P = 0.002).

**Table 2:** Correlations between IOP parameters and central corneal thickness parameters (Pearson correlation coefficient R and P value in parenthesis).

	CCT	CCET	CCST	CCSP
GAT IOP	0.648(<0.001)	0.057(0.696)	0.679(<0.001)	0.379(0.007)
NCT IOP	0.676(<0.001)	0.242(0.090)	0.678(<0.001)	0.199(0.165)
GAT IOP—NCT IOP	0.333(0.018)	0.435(0.002)	0.281(0.051)	-0.239(0.095)

GAT, Goldmann applanation tonometry; IOP, intraocular pressure; NCT, Non-contact tonometry; CCT, Central corneal thickness; CCET, Central corneal epithelial thickness; CCST, Central corneal stromal thickness; CCSP, Proportion of stromal thickness in central corneal thickness.

## Discussion

Although GAT is the standard and most commonly used method for measuring IOP, NCT is also commonly used in general health screenings because it can easily be used by non-medical personnel and there is a lower contamination risk than with GAT.[16] Both methods share the same disadvantage of error in the measured IOP value because both methods use applanation, which compresses the central part of the cornea. All forms of applanation are affected by biomechanical properties of cornea, including corneal stiffness, corneal thickness, scars of cornea, corneal surface irregularities, and corneal curvature.[17, 18]

IOPs measured using the two methods are known to correlate well and match in physiologically normal IOP ranges.[10, 11] However, some reports have revealed differences between the IOP measured via GAT and NCT in the same patients. Moseley et al.[19] compared GAT and NCT and they reported that NCT underestimated IOP at low pressures (<10 mmHg), while it tended to overestimate IOP at high pressures (>19 mmHg). Additionally, Tonnu et al.[12] and Zhang et el.[20] reported that NCT was more affected by CCT than GAT. In their study, the changes in the measured IOP for a 10-μm increase in CCT were 0.28 and 0.46 for GAT and NCT, respectively, showing that the IOP difference increased with an increasing CCT. Regine et al.[21] reported that the NCT IOP was higher than the GAT IOP (mean differences for right and left eye measurements were 1.37 and 1.17 mmHg, respectively), and NCT IOP showed a greater influence with corneal thickness. In our study, NCT IOP was higher than GAT IOP, and the difference between each tonometer increased with an increasing GAT IOP and thickening of CCT. Thus, we formulated the hypothesis that CCT is a major factor in the difference between the two tonometers based on this series of studies.

While an increasing CCT is associated with an artificially elevated IOP, increasing CCT due to corneal edema has been reported to make the measured IOP lower than the actual IOP value.[22, 23] However, in our clinical experience, we have observed that there is a great amount of variability in the relationship of edema and measured IOP, which was in fact one of the motivating factors for the current study. Histologically, the main layer of the corneal stroma comprises 60 to 70 successive layers made up of the lamellae of tightly bonded collagen fibers embedded in an extracellular matrix. There are variations in the direction of the collagen fibers in each layer to provide enough mechanical strength. When the lamellae are thicker and more abundant, more power is needed to compress a certain area of the cornea to be flattened; thus, the measured IOP values are necessarily increased. However, if there is corneal edema, water penetrates between and into the layered lamellae, and the corneal stroma swells like a sponge soaked with water; thus, less power is required to flatten the cornea, and the measured IOP values are necessarily decreased. Stelzer[24] explained that the area of applanation via a fixed force is greater in eyes with edematous corneas than in eyes without corneal edema, based on a study of enucleated edematous eyes.

The corneal epithelium is comprised of several cell layers, but unlike the stroma, lacks collagen fiber bundles. Thus, it would be assumed that the stroma is the primary contributor to corneal rigidity. In patients undergoing laser *in situ* keratomileusis (LASIK), a low residual stromal bed thickness is a significant risk factor for the development of ectasia after LASIK; additionally, the residual stromal bed thickness is more important than the residual total CCT in maintaining the integrity of the cornea.[25]

Based on the results of our study, which was conducted with normal patients without glaucoma medication and with no glaucomatous damage, the corneal thickness correlated significantly with NCT and GAT, and CCST showed a better correlation coefficient with the IOP than CCT. Additionally, CCET did not show a significant correlation with IOP. However, CCST, which represents the proportion of the corneal stroma within the full-thickness cornea, showed a significant positive correlation with GAT IOP but not with NCT IOP. Although it was not statistically significant, there was a tendency toward an increasing NCT IOP (but not GAT IOP) with a thickened CCET. When analyzing these results comprehensively, CCST showed a clear correlation with both NCT and GAT IOP, whreas the increase in CCET appeared to only slightly affect the increase in the NCT IOP measurement but not in the case of GAT IOP. This analysis was more pronounced when we evaluated the IOP difference between GAT and NCT. The IOP difference between the two tonometry methods increased significantly with thickening of the CCT, and thickening of the CCET. And CCET showed a greater effect on the IOP difference than the CCST.

In measuring IOP with NCT, the applanation force produced by the air puff increased linearly over 8 ms, progressively flattening the cornea, while the GAT measurement took longer because GAT directly compresses the cornea, and constant pressure should be applied until the examiner finds a balance point. Because the cornea exhibits a non-linear stress versus strain response with progressive stiffening at high strains, those differences in time duration of flattening forces might be a cause of the different relationships of NCT and GAT IOP with CCET and CCST.[26] Though speculative, we suspect that the combination of the differences in flattening force timing and structural differences of the epithelium and the stroma might explain why CCET was positively correlated with the IOP difference between GAT and NCT.

Our study did have limitations. First, it was designed as a cross sectional retrospective study; thus, we could not control all of the environmental factors, such as time, humidity, and temperature, while the patients were undergoing IOP and corneal thickness measurements. Second, the thickness measurement technique was a subjective method and we cannot guarantee that the exact CCST and CCT values were actually measured. To compensate for this obstacle we measured corneal thickness many times and if there was high variation in measured thickness then, further measurements were repeated. Additionally, the small number of patients may be another limitation of our study. However, each tonometry test was performed by the same examiner assigned to each piece of test equipment, and none of the patients had any other ocular pathological conditions. This is the first report of an analysis of the relationship in the differences between the two tonometry methods and corneal epithelial thickness.

In conclusion, both GAT IOP and NCT IOP increased with a thickened CCT, and CCST seemed to play a more important role than CCET. CCET also increased with a thickened CCT, which may be a possible explanation for the increasing difference in the IOP between GAT and NCT with an increasing CCT.

## Supporting Information

S1 Dataset Datasheet of involved patients. (XLSX)
